# Morpho-anatomical adaptations to waterlogging by germplasm accessions in a tropical forage grass

**DOI:** 10.1093/aobpla/plt047

**Published:** 2013-11-23

**Authors:** Juan Andrés Cardoso, Joisse Rincón, Juan de la Cruz Jiménez, Diana Noguera, Idupulapati M. Rao

**Affiliations:** 1 Centro Internacional de Agricultura Tropical (CIAT), Apartado Aéreo 6713, Cali, Colombia; 2 Programa de doctorado Biología Agraria y Acuicultura, Universidad de Granada, Avenida de Fuente Nueva s/n, Granada 18071, Spain; 3 BIO UPMC—Bioemco (UMR CNRS 7618), 32 Avenue Henri Varagnat, Bondy Cedex 93143, France; 4 Laboratori de Cartografia i SIG, Facultat de Geografia i Història, Universitat de Barcelona, Montalegre 6, Barcelona 08001, Spain

**Keywords:** Aerenchyma, *Brachiaria humidicola*, dry mass production, oxygen deficiency, root penetration, root traits.

## Abstract

Soil waterlogging reduces gas exchange between the soil and the atmosphere, leading to oxygen deprivation in the rhizosphere. *Brachiaria* spp. are the most widely sown forage grasses in tropical America. Among commercial *Brachiaria* grasses, *B. humidicola* shows superior tolerance to waterlogged soils based on maintenance of growth and reduced leaf chlorophyll loss and senescence. However, little is known about the underlying traits of waterlogging tolerance in *B. humidicola* or their intraspecific variation. For this purpose, an outdoor study was conducted using 12 germplasm accessions of *B. humidicola* that were grown in soil cylinders under drained or waterlogged soil conditions for 21 days. Dry mass production and morpho-anatomical responses (aerenchyma in shoots and roots, root diameter, proportional area of stele in roots, number of nodal and lateral roots, and length of the longest root) were determined. All accessions showed shorter roots and reduced root dry mass under waterlogged soil conditions. All accessions showed aerenchyma in shoots and roots under drained conditions but were further increased under waterlogging. All accessions showed a reduction in the proportional area of stele of roots in response to waterlogging. The accession (CIAT 26570) that showed a higher proportion of aerenchyma in shoots and roots and an increased number of nodal roots (with higher diameter and a reduction in the number of lateral roots) showed longer roots, less reduction in root dry mass and increased shoot growth under waterlogged conditions. We conclude that superior growth of one accession (CIAT 26570) under waterlogged soil conditions is probably a result of morpho-anatomical traits acting together to enhance root aeration and shoot ventilation. Further research is needed to test the ability to recover from waterlogging in *B. humidicola* accessions.

## Abstract (precis)

*Brachiaria humidicola*, a tropical forage grass, is recognized for its tolerance to temporary waterlogging. Waterlogged soils are characterized by slow movement of gases and oxygen defficiency. *B. humidicola* accessions showed adaptations common to wetland species, including a ventilation system (aerenchyma) from shoot to root that might facilitate O_2_ transport and the escape of gases that usually accumulate in roots under waterlogging. Of 12 accessions tested, one accession (CIAT 26570) showed greater aerenchyma formation. Quicker growth under waterlogging of CIAT 26570 might be associated with greater aerenchyma formation.

## Introduction

Soil waterlogging (or flooding of the soil) occurs when soil is saturated with water. Owing to the slow diffusion of gases in water, gas exchange between the soil and the atmosphere is strongly hindered (Colmer 2003). Soil waterlogging reduces plant growth as O_2_ availability in the root zone decreases (Armstrong 1979; Jackson and Drew 1984). To cope with waterlogging, plants usually develop new roots with aerenchyma (Laan *et al.* 1989; Visser *et al.* 1996; Huber *et al.* 2009). Aerenchyma refers to tissue with air spaces that provide an internal pathway for oxygen diffusion in organs under waterlogged/submerged conditions (Armstrong 1979). Apart from this, plants may display other adaptive strategies that might act together to improve root aeration and oxygen consumption within the root. These include increased root diameter (Armstrong 1979; Visser *et al.* 1997, 2000; Aguilar *et al.* 2008), fewer lateral roots developing from the main root axis (Armstrong *et al.* 1983; Sorrell *et al.* 2000; Aguilar *et al.* 2003) and narrower stele (Armstrong 1979; Armstrong and Beckett 1987; Armstrong *et al.* 2000; McDonald *et al.* 2002; Colmer 2003). Furthermore, to provide a continuum of gas ventilation between shoots and roots, aerenchyma is commonly present in shoots of plants that are well adapted to waterlogging (e.g. wetland species; Jackson and Armstrong 1999).

Soil waterlogging is a major limitation to pasture productivity (Hare *et al.* 2004). Perennial *Brachiaria* grasses (*Brachiaria* Trin. Griseb.) are the most widely sown forage grasses in tropical America (Miles *et al.* 2004; Valle and Pagliarini 2009). Among commercial *Brachiaria* grasses, *B. humidicola* tolerates the conditions of temporary waterlogged soils better than others based on a lower reduction in growth (Dias-Filho and Carvalho 2000; Silva *et al.* 2007; Caetano and Dias-Filho 2008), maintenance of leaf chlorophyll content and reduced leaf senescence (Dias-Filho and Carvalho 2000). *Brachiaria humidicola* is a highly stoloniferous grass that roots at each stolon node when in permanent contact with moist soil. This stoloniferous growth habit facilitates the spreading of *B. humidicola* over large areas (Canchila *et al.* 2011). However, a major limitation of *B. humidicola* is its low forage production and its moderate to low nutritional quality, which limits animal performance (Argel and Keller-Grein 1996). Variation in morphological traits (e.g. leaf area, leaf-to-stem ratio; Cruz 2009), forage production and protein concentration (Juarez *et al.* 2011) has been found among *B. humidicola* accessions. Furthermore, the discovery of a *B. humidicola* accession with a sexual mode of reproduction (Valle and Glienke 1991) has opened the opportunity to explore, through breeding, the genetic variation of *B. humidicola* (e.g. retain waterlogging tolerance while improving forage quality and yield).

Little is known of the underlying traits of waterlogging tolerance in *B. humidicola* or their intraspecific variation. Empirical observations in tropical forage grasses have associated waterlogging tolerance with a stoloniferous growth habit and the presence of hollow stolons (Baruch 1994). Preliminary research has shown that *B. humidicola* develops aerenchyma in roots under drained conditions and the trait is further increased under waterlogged conditions (Cardoso *et al.* 2010). Therefore, the main objective was to study in further detail some responses of *B. humidicola* accessions under waterlogged soil conditions*.* For this purpose we used a subset of 12 *B. humidicola* germplasm accessions from a total set of 66 accessions held in the gene bank of the International Center for Tropical Agriculture (CIAT) that in a preliminary study showed less damage in terms of leaf senescence and leaf chlorophyll content under waterlogged conditions (Rincón *et al.* 2010). We report differences in dry mass production and morpho-anatomical responses of shoots (aerenchyma in leaf sheaths and stolon internodes) and nodal roots (number of nodal roots, number of first-order lateral roots developed from nodal roots, root diameter, proportional areas of aerenchyma and stele, and length of the longest root) under drained or waterlogged soil conditions. These data will help to identify adaptive traits related to waterlogging tolerance in *B. humidicola*, and their variation, and therefore contribute to *B. humidicola* breeding.

## Methods

### Accessions and growing conditions

The genotypes used in this study were 12 germplasm accessions of *B. humidicola* (CIAT 679, CIAT 6013, CIAT 6133, CIAT 6707, CIAT 16182, CIAT 16866, CIAT 16886, CIAT 16888, CIAT 26152, CIAT 26181, CIAT 26416 and CIAT 26570). Three *Brachiaria* grasses with poor adaptation to waterlogged soil conditions (*B. brizantha* cv. Toledo, *B. ruziziensis* Br 44-02 and a *B.* hybrid cv. Mulato II) were included for reference (checks) but were excluded from the analysis.

The soil used in this study was an Oxisol collected from Santander de Quilichao, Department of Cauca in Colombia (latitude 3°60′N; longitude 76°310′W; altitude 990 m), 0–20 cm from the soil surface. All genotypes were grown from vegetative propagules. Propagules were harvested from 60-day-old plants growing in pots filled with 4 kg of a mixture of soil and sand (2 : 1) under field capacity and fertilized soil conditions (milligrams of element per kilogram of soil–sand mixture: N 21, P 26, K 52, Ca 56, Mg 15, S 10, Zn 1.0, Cu 1.0, B 0.05, Mo 0.05). Harvested propagules were then washed for 1 min in 0.1 % commercial sodium hypochlorite before planting. A 1 : 1 mixture of soil and sand was used to ensure better root growth and to facilitate root separation for analysis. Before planting, the soil mixture was thoroughly mixed with fertilizers. The rate of nutrient application (milligrams of element per kilogram of soil–sand mixture: N 40, P 50, K 100, Ca 101, Mg 28, S 20, Zn 2.0, Cu 2.0, B 0.1, Mo 0.1) represented the recommended fertility level for crop-pasture establishment (Rao *et al.* 1992). The soil mixture (4.5 kg) was packed in transparent plastic tubes (80 cm high × 7.5 cm diameter) inserted into opaque polyvinyl chloride (PVC) pipes. Three propagules of similar size (∼6 cm length) were planted 2 cm below the soil surface in each soil cylinder and thinned to one after rooting (5 days). Plants were allowed to grow for 21 days. Data on the number of fully expanded leaves, leaf greenness and maximum rooting depth (cm) were collected before the start of the experiment. Leaf greenness was measured in soil plant analysis development (SPAD) units in two fully expanded young leaves using a hand-held chlorophyll meter (SPAD-502, Konika Minolta, Japan). A factorial combination of 15 genotypes by two drainage conditions (drained or waterlogged) was established in a six-replicate randomized complete block. Waterlogging treatment was imposed by sealing the lower end of the PVC pipes with a cap and maintaining a water level of 3 cm above the soil surface. The soil of plants growing under drained soil conditions was kept at field capacity.

The experiment was conducted in an open area at the CIAT (Palmira, Colombia, latitude 3°31′N; longitude 76°19′W; altitude 965 m). During the experiment, temperature ran at an average of 30.5/23.8 °C (day/night), with a relative air humidity of 40.5/59.9 % (day/night) and a maximum photosynthetic photon flux density of 1800 µmol m^−2^ s^−1^. Redox potentials were monitored in four soil cylinders without plants during the experiment (two for drained soil, two for waterlogged soil) using a platinum electrode and a calomel reference electrode connected to a micro-voltmeter (ODR meter, Eijkelkamp, The Netherlands). Redox potentials of drained soil remained stable around 450 mV. Soil redox potential decreased to values below 330 mV after 7 days of waterlogging treatment and stabilized at around 100 mV after 14 days.

### Harvest

Prior to harvesting, leaf greenness (SPAD) and maximum rooting depth (cm) for each experimental unit (plant) were assessed. SPAD values were recorded in the same leaves used for greenness determination before the start of the experiment. Maximum rooting depth was estimated in roots that looked white and healthy and that were growing next to the wall of the transparent cylinders. Plants were harvested after 21 days of growth under drained or waterlogged soil conditions. Shoots were cut at 3 cm above the soil and gently washed with running water. Leaves, stolons (runners) and tillers (erect culms) (these three representing the shoot) were separated and oven dried for 72 h at 60 °C for determination of dry mass. Fully expanded leaves were counted before dry mass determination.

The remaining parts (underground parts and 3 cm above the soil) were washed from soil with running water and placed in a container with a few drops of low-foaming detergent (polysorbate 20) for 10–15 min and rinsed again with tap water to clean up loosened soil. After washing, parts were kept in 50 % ethanol solution and stored at 4 °C for later separation and analysis.

### Morphology and anatomy of nodal roots

Parts kept in 50 % ethanol solution were further separated. Roots were separated from shoots. Under a dissecting microscope, roots that appeared damaged were sorted out and discarded. After that, the number of nodal roots was counted. To confirm the maximum rooting depth in soil, the length of the longest nodal root (waterlogged only) for each plant was recorded. After that and for each plant, a sample of four nodal roots of 11–14 cm length was taken. These roots were placed in an acrylic tray filled with water and carefully arranged so that there was no overlapping of lateral roots. These roots were scanned to record grey images at 600 dpi with a dual scanner (EPSON Expression 1680, Japan). Scanned images were then used for manual determination of the number of lateral roots (first order only) developed from the main axis of nodal roots.

Roots employed for determination of the number of lateral roots were used in the measurement of the diameter and areas of aerenchyma and stele of nodal roots. The diameter and areas of aerenchyma and stele were measured in cross-sections taken at 2.0, 5.0, 10 and 11–14 cm behind the root tip. Sections of roots were taken using a hand-held razor blade and examined under a light microscope that was equipped with a digital camera (Nikon Coolpix 4500, Japan). An eyepiece reticle was used to record root diameters. The areas of aerenchyma and stele (expressed as a percentage of total cross-sectional area) were determined in each digital picture using ImageJ software (v. 1.38, National Institutes of Health, USA). Cross-sections of first-order lateral roots were also taken when possible.

### Areas of aerenchyma in leaf sheaths and internodes of stolons

Stolon pieces (3–5 cm length) kept in 50 % ethanol solution were used for determination of aerenchyma in leaf sheaths and internodes of stolons. The greenest stolon per plant was visually selected, and two to five cross-sections of leaf sheaths and internodes were taken at 1 cm from the first observable node from the root–shoot junction. The areas of aerenchyma in each cross-section were analysed as described above.

Once morpho-anatomical traits had been recorded, the remaining parts kept in 50 % ethanol were used for determination of dry weight. As cross-sections used for microscopy studies did not exceed 3 % of fresh weight, the remaining non-cross-sectioned parts were also taken into account for dry weight determination. The remaining shoots and roots were oven dried at 60 °C for 72 h for dry mass determination.

### Statistical analysis

Data were analysed to generate mean values, standard deviation and analysis of variance (ANOVA) using R software (v. 2.15.2) (R Development Core Team, 2012). Log transformation was carried out to ensure normality of data. Differences between accessions were analysed and reported with the least significant difference (LSD) at *α* = 0.05.

## Results

### Effect of waterlogging on leaf number, leaf greenness and dry mass production

**Figure 1.**
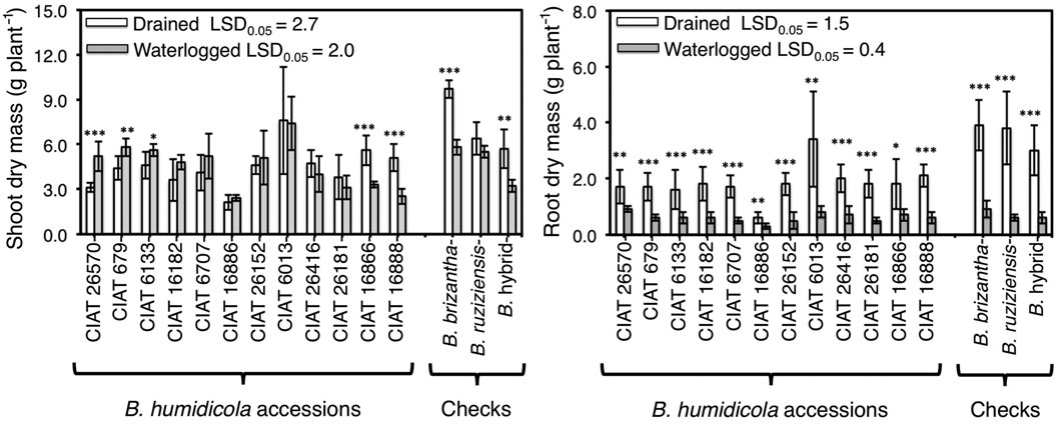
Shoot and root dry mass of 12 *B. humidicola* accessions (plus three checks: *B. brizantha*, *B. ruziziensis* and *B.* hybrid) grown under drained (white column) or waterlogged soil (grey column) conditions for 21 days. Columns represent means and error bars their standard deviation (*n* = 6). An asterisk represents significant differences between treatments for each accession (statistical significance at the *0.05, **0.01 and ***0.001 probability levels). LSD values exclude checks.

Prior to the beginning of the experiment, all accessions showed a similar number of leaves per plant (around 14 leaves). Leaf production increased in all accessions when grown under drained or waterlogged soil conditions (*P* < 0.05), but CIAT 6013 produced more leaves than the rest of the accessions tested (Table 1). There were no significant differences in leaf production among plants grown under waterlogging when compared with plants grown under drained soil conditions (Table 1). SPAD values decreased in all accessions of *B. humidicola* from the beginning of the experiment under drained or waterlogged soil conditions (*P* < 0.05), but these values were not further reduced under waterlogging **[see** **Supporting Information****]**. When compared with the drained treatment, only CIAT 26570, CIAT 679 and CIAT 6133 increased their shoot dry mass, whereas CIAT 16866 and CIAT 16888 showed a reduction of shoot dry mass under waterlogged conditions (Fig. 1). Waterlogging treatment significantly reduced root dry mass in all accessions (Fig. 1). Data shown for shoot dry mass are the sum of leaves, stolons and tillers, but there was a trend to allocate more biomass to stolons **[see** **Supporting Information****]**.

**Table 1.** Number of leaves of 12 *B. humidicola* accessions (plus three checks: *B. brizantha*, *B. ruziziensis* and *B.* hybrid) before the beginning of the experiment and after 21 days of growth under drained or waterlogged soil. Data shown are means of six replicates ± SD. An asterisk represents significant differences between treatments for each accession (statistical significance at the *0.05, **0.01 and ***0.001 probability levels). All accessions showed an increase in the number of leaves under both treatments when compared with initial values (*P* < 0.05). NS: not significant. *P*_anova_ and LSD values exclude checks.

Accession	Number of leaves (plant^−1^)			
	0 days of treatment		21 days of treatment	
	Drained	Waterlogged	Drained	Waterlogged
CIAT 26570	13.7 ± 5.1	14.3 ± 5.7	56.5 ± 20.1	72.0 ± 23.2
CIAT 679	13.8 ± 6.0	12.7 ± 4.8	39.5 ± 16.8	40.0 ± 16.2
CIAT 6133	13.5 ± 4.6	14.3 ± 5.6	33.8 ± 11.5	38.0 ± 11.6
CIAT 16182	15.8 ± 7.8	14.8 ± 9.4	47.2 ± 19.6	38.5 ± 16.9
CIAT 6707	14.0 ± 2.8	12.7 ± 5.1	39.0 ± 11.0	38.3 ± 12.0
CIAT 16886	10.3 ± 4.3	14.3 ± 3.9	32.5 ± 17.8	51.2 ± 30.2
CIAT 26152	17.0 ± 5.2	14.0 ± 4.0	76.3 ± 31.8	52.0 ± 16.1
CIAT 6013	16.7 ± 5.9	18.5 ± 5.0	122.7 ± 59.2	82.5 ± 30.7
CIAT 26416	12.5 ± 7.2	12.0 ± 4.0	49.0 ± 9.9	39.8 ± 14.5
CIAT 26181	12.2 ± 3.8	10.3 ± 5.3	45.5 ± 28.1	25.0 ± 14.8
CIAT 16866	16.3 ± 8.1	14.7 ± 4.1	46.8 ± 27.6	37.3 ± 9.2
CIAT 16888	11.0 ± 3.1	12.0 ± 4.1	56.3 ± 31.4	28.0 ± 5.4
*P*_anova_	0.5159	0.5517	0.0000	0.0000
LSD_0.05_	NS	NS	51.4	40.7
Checks				
*B. brizantha*	11.2 ± 3.4	9.8 ± 1.9	21.3 ± 8.5	7.0 ± 2.4**
*B. ruziziensis*	16.0 ± 5.7	15.0 ± 5.9	42.2 ± 5.8	10.0 ± 3.5**
*B*. hybrid	9.0 ± 3.5	9.8 ± 3.0	33.7 ± 5.3	7.0 ± 1.7**

### Effect of waterlogging on root morphology and anatomy

Waterlogging increased the number of nodal roots per plant in six accessions (CIAT 16888, CIAT 16886, CIAT 6133, CIAT 6707, CIAT 679 and CIAT 26570; Table 2). Nodal roots formed under waterlogged conditions were almost invariably whiter than roots of plants grown under drained conditions (visual observation). Most of the nodal roots developed fewer lateral roots (first order) when grown under waterlogged conditions; however, this was only statistically significant for seven accessions (CIAT 16888, CIAT 26416, CIAT 6013, CIAT 6133, CIAT 6707, CIAT 679 and CIAT 26570; Table 2). Prior to the beginning of the experiment, the maximum rooting depth of all accessions was around 25 cm below the soil surface **[see** **Supporting Information****]**. When grown under drained soil conditions, nodal roots of all accessions reached the bottom of the cylinders (77 cm) prior to the completion of the experiment. Under waterlogged conditions, the maximum rooting depth of all accessions did not appear to grow deeper than 30 cm below the soil surface. This was confirmed at harvest with measurements of the length of the longest nodal root (Table 2). Among *B. humidicola* accessions, CIAT 26152 showed the shortest nodal roots (15.5 cm) whereas roots of CIAT 26570 were the longest (26.4 cm) under waterlogged soil conditions (Table 2).

**Table 2.** Number of nodal and lateral roots and length of the longest nodal root (LLN) of 12 *B. humidicola* accessions (plus three checks: *B. brizantha*, *B. ruziziensis* and *B.* hybrid) grown under drained or waterlogged soil for 21 days. The number of lateral roots (first order only) was determined from nodal roots of 11–14 cm length. Data shown are means of six replicates ± SD. An asterisk represents significant differences between treatments for each accession (statistical significance at the *0.05, **0.01 and ***0.001 probability levels). *P*_anova_ and LSD values exclude checks.

Accession	Number of nodal roots (plant^−1^)		Number of lateral roots		LLN (cm plant^−1^)
	Drained	Waterlogged	Drained	Waterlogged	Waterlogged
CIAT 26570	21.2 ± 3.5	26.5 ± 4.1*	10.5 ± 0.8	7.0 ± 0.7***	26.4 ± 5.6
CIAT 679	17.0 ± 3.9	36.0 ± 8.0***	10.8 ± 1.4	5.6 ± 0.5***	21.0 ± 3.7
CIAT 6133	20.0 ± 4.7	31.3 ± 7.3**	9.2 ± 1.1	7.8 ± 1.0*	23.2 ± 3.2
CIAT 16182	22.8 ± 6.9	28.8 ± 7.4	10.0 ± 1.5	9.6 ± 3.0	19.3 ± 3.0
CIAT 6707	20.0 ± 2.6	27.7 ± 6.3*	10.1 ± 1.4	6.2 ± 0.9***	22.2 ± 4.8
CIAT 16886	20.5 ± 8.5	37.3 ± 3.1**	10.3 ± 1.6	9.0 ± 2.3	17.2 ± 4.4
CIAT 26152	37.0 ± 10.8	39.3 ± 13.6	8.8 ± 1.2	8.0 ± 1.7	15.5 ± 2.6
CIAT 6013	42.7 ± 10.7	31.8 ± 9.4	10.2 ± 1.0	8.4 ± 1.5*	21.8 ± 4.8
CIAT 26416	41.5 ± 9.3	34.0 ± 4.5	11.9 ± 1.3	6.4 ± 1.7***	20.0 ± 3.3
CIAT 26181	26.7 ± 8.1	34.0 ± 5.2	6.8 ± 0.6	7.6 ± 1.5	18.0 ± 1.8
CIAT 16866	26.5 ± 4.1	34.8 ± 8.9	7.1 ± 1.1	7.8 ± 1.7	19.7 ± 4.4
CIAT 16888	42.7 ± 3.1	53.7 ± 10.7*	9.1 ± 1.1	6.3 ± 0.7***	17.0 ± 2.4
*P*_anova_	0.0000	0.0000	0.0000	0.001	0.000
LSD_0.05_	13.3	15.0	2.3	3.0	7.3
Checks					
*B. brizantha*	33.2 ± 4.6	30.3 ± 3.1	6.6 ± 1.4	5.5 ± 1.2	12.5 ± 2.7
*B. ruziziensis*	49.2 ± 2.3	48.8 ± 9.1	9.0 ± 1.0	8.5 ± 2.4	12.5 ± 1.9
*B*. hybrid	46.3 ± 8.9	34.0 ± 4.1*	5.3 ± 1.2	4.4 ± 1.3	9.3 ± 2.0

**Figure 2.**
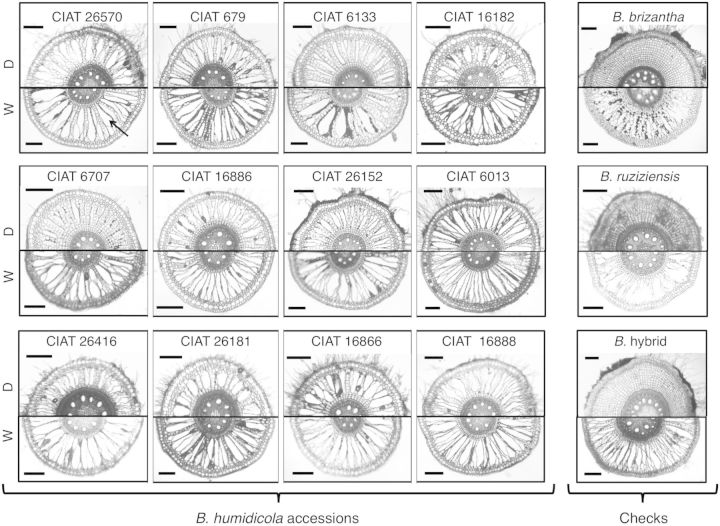
Aerenchyma development in nodal roots of 12 *B. humidicola* accessions (plus three checks: *B. brizantha*, *B. ruziziensis* and *B.* hybrid) grown under drained (D) or waterlogged (W) soil conditions for 21 days. Cross-sections taken at 10 cm from the root tip. The arrow indicates an air space in the cortex. Scale bars = 250 µm.

**Figure 3.**
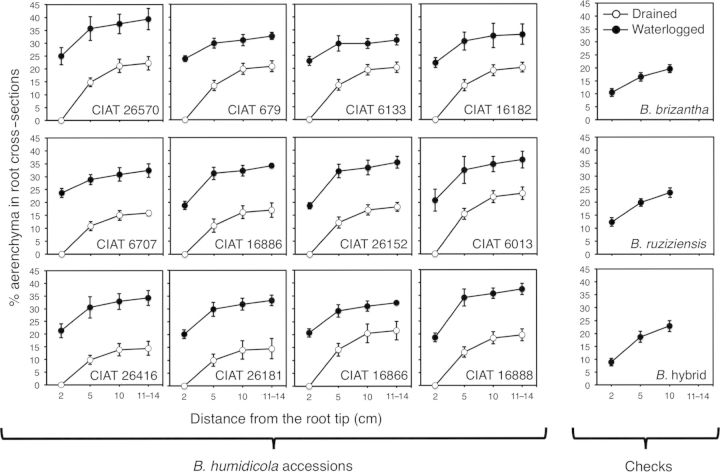
Percentage of aerenchyma along nodal roots of 12 *B. humidicola* accessions (plus three checks: *B. brizantha*, *B. ruziziensis* and *B.* hybrid) grown under drained (open circles) or waterlogged soil (closed circles) conditions for 21 days. Circles represent means and error bars their standard deviation (*n* = 6). Significant differences at *P* < 0.01 for treatments at each root distance were found.

Nodal roots grown under waterlogged soil tended to be of greater diameter, but only seven accessions (CIAT 16888, CIAT 26181, CIAT 26416, CIAT 6133, CIAT 6707, CIAT 679 and CIAT 26570) showed significant increases when compared with nodal roots of drained plants (Table 3). Almost invariably, nodal roots of waterlogged plants lacked root hairs (visual observation; Fig. 2). Aerenchyma developed in nodal roots of all accessions of *B. humidicola* grown under drained soil conditions (Fig. 2). The extent of aerenchyma development under drained conditions varied among accessions (Figs 2 and 3, Table 3). Growth in waterlogged soil increased aerenchyma development and decreased the relative proportion of stele in cross-sections of nodal roots in all accessions tested (Fig. 2, Table 3). For all accessions, the relative proportions of aerenchyma and stele along the root length decreased from the root base to the root tip (Fig. 3; data not shown for stele). Aerenchyma development in nodal roots was absent at sites of the presence and/or emergence of lateral roots (visual observation) **[see** **Supporting Information****]**. Lateral roots showed negligible aerenchyma and their diameters were 10–30 times smaller than those of nodal roots (visual observation).

**Table 3.** Average diameter, aerenchyma and stele of nodal roots of 12 *B. humidicola* accessions (plus three checks: *B. brizantha*, *B. ruziziensis* and *B.* hybrid) grown under drained or waterlogged soil for 21 days. Data shown are means of six replicates ± SD. An asterisk represents significant differences between treatments for each accession (statistical significance at the *0.05, **0.01 and ***0.001 probability levels). *P*_anova_ and LSD values exclude checks.

Accession	Diameter (mm)		% aerenchyma		% stele	
	Drained	Waterlogged	Drained	Waterlogged	Drained	Waterlogged
CIAT 26570	1.5 ± 0.2	1.8 ± 0.1**	14.5 ± 1.7	34.4 ± 3.8***	15.0 ± 1.1	6.4 ± 0.8***
CIAT 679	1.4 ± 0.1	1.7 ± 0.1***	13.5 ± 1.2	29.3 ± 1.6***	16.9 ± 2.9	8.1 ± 0.8***
CIAT 6133	1.5 ± 0.0	1.7 ± 0.0***	13.3 ± 1.5	28.3 ± 2.0***	16.6 ± 1.9	8.2 ± 0.9***
CIAT 16182	1.1 ± 0.1	1.2 ± 0.3	13.2 ± 1.5	29.6 ± 3.5***	11.3 ± 0.9	6.9 ± 0.8***
CIAT 6707	1.0 ± 0.2	1.4 ± 0.1**	10.4 ± 1.0	28.9 ± 2.2***	14.8 ± 2.2	6.7 ± 0.2***
CIAT 16886	1.2 ± 0.1	1.1 ± 0.3	11.0 ± 1.9	31.5 ± 2.1***	15.0 ± 2.7	7.6 ± 0.3***
CIAT 26152	1.4 ± 0.1	1.5 ± 0.3	11.8 ± 1.4	29.8 ± 2.1***	12.5 ± 1.2	7.4 ± 0.7***
CIAT 6013	1.6 ± 0.3	1.9 ± 0.3	15.3 ± 1.7	31.2 ± 3.8***	15.2 ± 2.2	9.2 ± 1.4***
CIAT 26416	1.1 ± 0.1	1.5 ± 0.1***	9.5 ± 1.7	29.7 ± 3.0***	23.8 ± 1.9	9.5 ± 1.5***
CIAT 26181	1.2 ± 0.2	1.7 ± 0.1***	9.6 ± 2.5	28.8 ± 1.9***	12.5 ± 3.0	7.3 ± 1.6**
CIAT 16866	1.2 ± 0.1	1.3 ± 0.3	14.0 ± 2.4	28.3 ± 1.3***	14.2 ± 1.9	7.0 ± 0.6***
CIAT 16888	1.5 ± 0.0	1.6 ± 0.1*	12.8 ± 1.7	31.5 ± 2.1***	12.6 ± 0.2	7.4 ± 1.5***
*P*_anova_	0.0000	0.0000	0.0000	0.01	0.0000	0.0000
LSD_0.05_	0.3	0.4	3.3	4.9	3.8	2.0
Checks						
*B. brizantha*	1.9 ± 0.1	2.0 ± 0.1	–	15.6 ± 1.4	17.8 ± 1.7	14.9 ± 2.8
*B. ruziziensis*	1.1 ± 0.1	1.3 ± 0.2	–	18.3 ± 1.3	19.2 ± 2.5	17.1 ± 1.1
*B*. hybrid	2.0 ± 0.1	1.7 ± 0.1***	–	16.8 ± 1.5	19.1 ± 1.7	16.2 ± 1.1**

### Effect of waterlogging on proportional areas of aerenchyma in leaf sheaths and internodes of stolons

**Figure 4.**
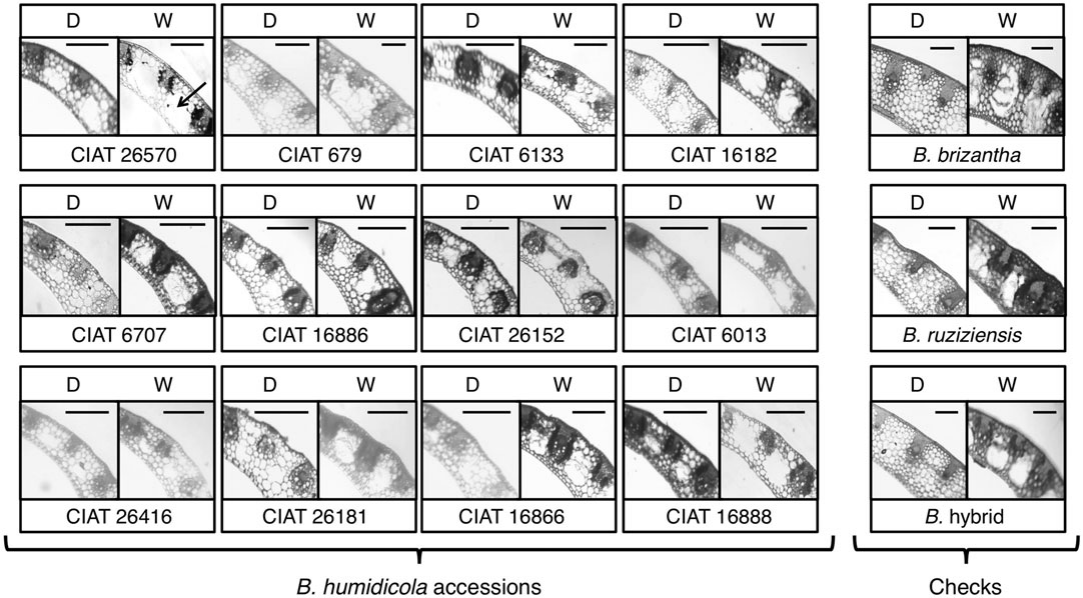
Aerenchyma development in leaf sheaths of 12 *B. humidicola* accessions (plus three checks: *B. brizantha*, *B. ruziziensis* and *B.* hybrid) grown under drained (D) or waterlogged (W) soil conditions for 21 days. Cross-sections taken at 1 cm from the first observable node from the root–shoot junction. An arrow indicates air spaces in the leaf sheath. Scale bars = 500 µm.

**Figure 5.**
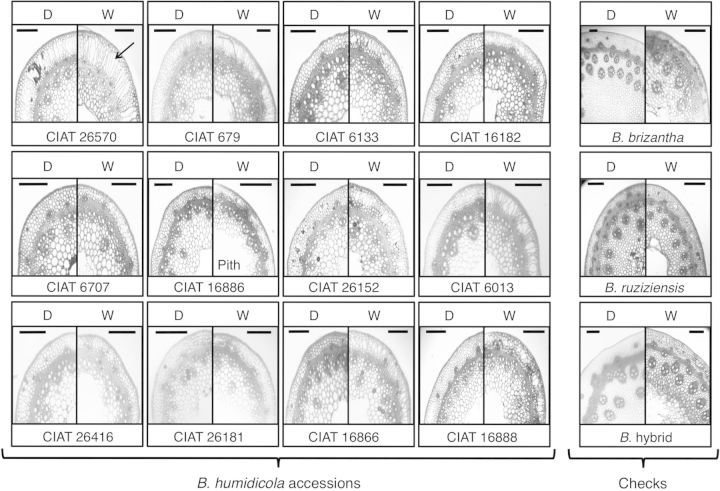
Aerenchyma development in stolon internodes of 12 *B. humidicola* accessions (plus three checks: *B. brizantha*, *B. ruziziensis* and *B.* hybrid) grown under drained (D) or waterlogged (W) soil conditions for 21 days. Cross-sections taken at 1 cm from the first observable node from the root–shoot junction. The arrow indicates an air space in the cortex of stolons. All stolons of *B. humidicola* grown either under drained or waterlogged conditions showed a hollowed pith. Scale bars = 500 µm.

In all *B. humidicola* accessions, aerenchyma developed in leaf sheaths, the pith cavity and in the cortex of stolon internodes even under drained conditions (Figs 4 and 5, Table 4). Leaf sheaths and stolon internodes in all accessions showed increased aerenchyma development under waterlogged conditions (Figs 4 and 5, Table 4). Aerenchyma was absent in nodes.

**Table 4.** Percentage of aerenchyma in shoots (leaf sheaths and stolon internodes) of 12 *B. humidicola* accessions (plus three checks: *B. brizantha*, *B. ruziziensis* and *B.* hybrid) grown under drained or waterlogged soil for 21 days. Data shown are means of six replicates ± SD. An asterisk represents significant differences between treatments for each accession (statistical significance at the *0.05, **0.01 and ***0.001 probability levels). *P*_anova_ and LSD values exclude checks.

Accession	% aerenchyma in shoot					
	Leaf sheaths		Pith (stolon internode)		Cortex (stolon internode)	
	Drained	Waterlogged	Drained	Waterlogged	Drained	Waterlogged
CIAT 26570	4.9 ± 0.7	10.3 ± 0.7***	3.3 ± 1.6	7.7 ± 1.2***	7.8 ± 0.7	18.4 ± 1.7***
CIAT 679	4.2 ± 0.8	8.8 ± 0.8***	5.1 ± 0.8	7.6 ± 1.3**	4.5 ± 1.5	9.2 ± 1.5***
CIAT 6133	4.0 ± 0.9	7.4 ± 0.7***	4.7 ± 1.0	7.1 ± 1.4**	1.3 ± 0.5	6.8 ± 0.7***
CIAT 16182	4.3 ± 0.5	7.7 ± 0.7***	2.8 ± 0.7	4.9 ± 0.9**	3.3 ± 0.9	7.1 ± 1.5***
CIAT 6707	3.6 ± 0.5	7.8 ± 0.8***	2.3 ± 0.6	5.2 ± 1.1***	1.3 ± 0.8	5.9 ± 0.8***
CIAT 16886	2.7 ± 0.5	5.6 ± 0.5***	7.2 ± 0.8	14.6 ± 1.4***	1.2 ± 0.6	5.9 ± 1.5***
CIAT 26152	3.5 ± 0.7	6.4 ± 0.8***	2.4 ± 0.6	4.5 ± 1.0**	3.2 ± 0.5	8.1 ± 0.7***
CIAT 6013	4.3 ± 0.7	10.2 ± 0.8***	4.4 ± 1.4	8.0 ± 1.3**	1.9 ± 0.4	10.7 ± 2.2***
CIAT 26416	3.8 ± 0.7	6.2 ± 0.6***	3.0 ± 0.8	6.1 ± 0.9***	3.4 ± 1.5	7.9 ± 1.3***
CIAT 26181	3.1 ± 0.5	6.9 ± 0.7***	5.2 ± 1.3	9.1 ± 1.8**	1.6 ± 0.5	7.8 ± 1.8***
CIAT 16866	3.8 ± 0.5	7.3 ± 1.0***	4.0 ± 0.8	7.5 ± 1.1***	2.7 ± 0.6	6.9 ± 1.5***
CIAT 16888	5.3 ± 0.3	8.5 ± 0.6***	5.3 ± 0.7	7.8 ± 1.2**	2.9 ± 1.2	5.9 ± 1.3***
*P* value	0.0000	0.0000	0.0000	0.0000	0.0000	0.0000
LSD_0.05_	1.2	1.4	2.2	2.4	1.7	2.7
Checks						
*B. brizantha*	–	6.5 ± 0.6	–	5.2 ± 1.9	–	–
*B. ruziziensis*	–	7.0 ± 0.7	–	5.5 ± 1.3	–	–
*B*. hybrid	–	6.3 ± 0.9	–	4.6 ± 2.0	–	–

## Discussion

In the present study, an increase in the number of leaves in all *B. humidicola* accessions was an indication that plants kept growing under waterlogging, even if some accessions showed reductions of shoot dry mass under this condition when compared with the drained treatment (Fig. 1). It was noteworthy that one accession, CIAT 6013, was very productive under both drained and waterlogged soil conditions (Fig. 1). The high production of leaves from the start of the experiment together with the higher production of shoot dry mass of CIAT 6013 suggest a faster growth rate than the rest of the tested accessions. From an agronomic point of view, CIAT 6013 shows promise for use in zones with a risk of waterlogging events. However, the inherent high productivity of CIAT 6013 may not be in itself causally related to waterlogging tolerance since it has been shown in other grasses (e.g. wheat, barley and oats) that vigour and tolerance are not necessarily positively linked (Setter and Waters 2003; Pang *et al.* 2004).

The reduction in growth of root mass in water-saturated soils is common in flood-tolerant grasses (Naidoo and Naidoo 1992; Baruch 1994; Loreti and Oesterheld 1996). Waterlogging-induced new root formation is a well-documented response that presumably allows the plant to compensate for the decay of the original root system brought about by a lack of oxygen (Jackson and Drew 1984; Vartapetian and Jackson 1997). Therefore, increasing the number of nodal roots is considered of high importance for survival under waterlogged conditions (Visser *et al.* 1996). In the present study, six accessions showed an increase in the number of nodal roots under waterlogged conditions (Table 2). However, none of the remaining accessions showed a reduction in the number of nodal roots under waterlogged conditions when compared with plants grown under drained soil (Table 2). Therefore, it is likely that a reduction of root dry mass in all accessions was mainly a reflection of the restriction of root penetration below 30 cm soil depth.

Roots can display various adaptations to waterlogged soils; among them, the presence of aerenchyma is the most obviously adaptive one (Laan *et al.* 1989). All accessions of *B. humidicola* developed aerenchyma in nodal roots even under well-drained soil, suggesting that this is a constitutive trait in this species (Figs 2 and 3, Table 3). Constitutive root aerenchyma is common in plants adapted to waterlogged conditions (Justin and Armstrong 1987; Jackson and Armstrong 1999; Visser *et al.* 2000; Mano *et al.* 2006; Abiko *et al.* 2012). As development of aerenchyma takes time, constitutive root aerenchyma would aid root aeration during the initial stages of waterlogging (Mano *et al.* 2006; Colmer and Voesenek 2009; Abiko *et al.* 2012). Overall aerenchyma in cross-sections of nodal roots under drained conditions in *B. humidicola* accessions (14–24 %) was similar to values found in other grass species that are adapted to waterlogging such as *Oryza sativa* (Armstrong 1971; Colmer *et al.* 1998), *Glyceria maxima* (Smirnoff and Crawford 1983), *Brachiaria mutica* and *Echinochloa polystachya* (Baruch and Merida 1995), which typically range from 20 to 25 % under O_2_-sufficient conditions (McDonald *et al.* 2001). The area of aerenchyma in roots of *B. humidicola* accessions increased over 2-fold from constitutive levels when grown in waterlogged soil (Table 3). This finding is similar to the 1.5- to 3.0-fold increase reported for several wetland grasses grown in flooded compared with drained sand (Smirnoff and Crawford 1983). In the present study, the accessions that showed greater levels of root aerenchyma under drained conditions did not necessarily show greater aerenchymatous tissue under waterlogged soils, as was shown in other species (e.g. *Zea nicaraguensis*; Mano and Omori 2013). This indicates that high constitutive aerenchyma alone may not be a good indicator of the adaptation ability to transient waterlogging among *B. humidicola* accessions and, therefore, evaluation of aerenchyma development under this soil condition should be taken into account.

An increase in root thickness will allow the plant to cope better with waterlogged soils as it contributes to an increase in O_2_ flow to the root tip (Armstrong 1979; Aguilar *et al.* 2008). Seven accessions showed this morphological acclimation (Table 3). An increase in root diameter under waterlogged conditions was previously reported in other tropical grasses (Baruch and Merida 1995; Imaz *et al.* 2012). The development of nodal roots with a larger diameter may increase the cortex area in which aerenchyma can develop and would also help in ventilating gases such as ethylene and carbon dioxide, which typically increases under waterlogging conditions (Visser *et al.* 1997, 2000).

The proportional area of stele in *B. humidicola* accessions decreased by 39–60 % under waterlogging (Table 3). This morphological acclimation was also documented in other species such as *Hyparrhenia ruffa* (34 % reduction; Baruch and Merida 1995) and *Hordeum vulgare* (20 % reduction; Pang *et al.* 2004). Studies on *Zea mays* (Armstrong 1994) and *Musa* sp. (Aguilar *et al.* 2003) found that the respiratory demands of stele are about four to six times greater than those found in the cortex; thus a useful adaptation to restricted O_2_ supply might be a narrower stele (Armstrong *et al.* 2000). A narrower stele would allow less consumption of O_2_ per unit length of the diffusion path of the aerenchyma that leads to the root tip (Armstrong *et al.* 2000) and would also allow a greater cortex where aerenchyma can be formed (Justin and Armstrong 1987; McDonald *et al.* 2002).

A reduction in the number of lateral roots under oxygen-deficient conditions might be beneficial as each lateral root demands oxygen for growth and maintenance, thereby reducing transport of O_2_ along the root where they develop and therefore to the root tip (Armstrong *et al.* 1983; Aguilar *et al.* 2003). The reduction in the number of lateral roots under waterlogging, as shown by seven accessions (Table 2), was also recorded for other species such as *Pisum sativum* (Armstrong *et al.* 1983) and *Musa* sp. (Aguilar *et al.* 2003). Furthermore, development of lateral roots seemed to locally restrict aerenchyma development in the nodal roots of *B. humidicola* **[see Supporting Information]**. Lack of aerenchyma adjacent to lateral root emergence was also noted by other authors (Smirnoff and Crawford 1983; Justin and Armstrong 1991; Enstone and Peterson 2005). As lateral roots develop from the pericycle, their presence occupies space that would otherwise be aerenchymatous (Justin and Armstrong 1991).

Reduction of root penetration from the beginning of waterlogging coincides with depletion of O_2_ as shown by redox potential values <330 mV (where molecular oxygen is undetectable; Ponnamperuma 1972) after 7 days. Roots stop growing if the root tip becomes anoxic (Armstrong and Drew 2002). As root penetration into anaerobic soil relies greatly upon the capacity for internal O_2_ transport to the root tip (for a review see Colmer 2003; Colmer and Greenway 2005), greater aerenchyma formation, as shown by CIAT 26570, would facilitate deeper penetration of roots into waterlogged soil.

Aerenchyma in the shoot provides a ventilation channel between shoots and submerged roots (Sorrell *et al.* 1994; Colmer 2003; Afreen *et al.* 2007; Armstrong *et al.* 2009). Although aerenchyma was absent in the nodes of *B. humidicola*, it is possible that the continuum of the ventilation path between shoots and roots was restricted (rather than impeded) to intercellular spaces in the nodes, as was suggested in rice (Butterbach-Bahl *et al.* 1997; Steffens *et al.* 2011). In this sense, the presence of a ventilation channel in leaf sheaths and stolon internodes (Figs 4 and 5, Table 4) might also contribute to the good adaptation of *B. humidicola* to waterlogged soils by enabling O_2_ transport from shoot to root, but also by facilitating the escape of gases (such as both carbon dioxide and ethylene which usually accumulate in the roots under waterlogged soil conditions; Visser *et al.* 1997). Very interestingly, CIAT 26570 also showed more aerenchyma in leaf sheaths and internodes than the rest of the accessions tested (Figs 4 and 5, Table 4).

## Conclusions

The results obtained from this study help to identify adaptive traits associated with waterlogging tolerance in *B. humidicola*. Constitutive aerenchyma in nodal roots may allow *B. humidicola* accessions to tolerate oxygen deficiency from the onset of waterlogging. During waterlogging, development of more and thicker nodal roots with a higher proportion of aerenchyma, smaller stele area and showing a reduction of lateral roots may act together for a more efficient system for O_2_ transport to the elongation zone of the root (cf. Colmer 2003; cf. Colmer and Greenway 2005). The accession that showed this combination of root traits (CIAT 26570) showed larger roots (Table 3) and less reductions of root dry mass under waterlogged conditions (Table 1). Furthermore, the increase of aerenchyma in leaf sheaths and internodes above constitutive levels might improve the ventilation of gases between roots and shoots under waterlogged soil conditions. A more extensive root aeration system combined with a more prominent ventilation system in the shoot would probably allow CIAT 26570 to grow more quickly under waterlogged conditions than the other accessions we tested.

Evaluation of plants for their tolerance to waterlogging should also consider their ability to recover after the soil drains (Malik *et al.* 2002; Zhou 2010; Bramley *et al.* 2011; Striker 2012). For example, genotypes may differ in their responses to waterlogging but their variation after subsequent drainage might be even more marked (e.g. cool-season grain legumes; Solaiman *et al.* 2007). Morpho-anatomical changes of the root might be beneficial for growth under waterlogging but may bring up limitations for growth under non-waterlogged conditions (McDonald *et al.* 2002). For example, reduced stele size was associated with smaller meta-xylem vessels in *Alisma triviale*, which in turn might be responsible for reduced hydraulic conductivity (Ryser *et al.* 2011). Furthermore, waterlogging significantly reduces root penetration, and thereby resumption of deep root growth may be of great importance for exploration of soil nutrients and water after the waterlogged soil has been drained (Thomson *et al.* 1992). In this sense, aspects of root function and growth after waterlogging, and whether tolerance of *B. humidicola* accessions during waterlogging translates into the ability to grow after subsequent soil drainage, should be taken into consideration in future studies.

## Sources of Funding

This work was partially funded by Fontagro (USA) (project number: FTG-8060/08).

## Contributions by the Authors

J.A.C. was involved in designing the experiments, data collection and analysis, manuscript preparation and submission. J.R. contributed to the set-up of experiments. J.C.J. and D.N. provided technical assistance during experiments. I.M.R. was involved in designing and supervision of experiments, manuscript preparation and submission.

## Conflicts of Interest Statement

None declared.

## Supporting Information

The following Supporting Information is available in the online version of this article –

**File 1.** Table. Changes in leaf greenness (SPAD units) of 12 *B. humidicola* accessions (plus three checks: *B. brizantha*, *B. ruziziensis* and *B.* hybrid) grown under drained or waterlogged soil for 21 days. Data shown are means of six replicates ± SD. An asterisk represents significant differences between treatments for each genotype (statistical significance at the *0.05, **0.01 and ***0.001 probability levels). All accessions showed a decrease in SPAD values under both treatments when compared with initial values (*P* < 0.05). *P*_anova_ and LSD values exclude checks.

**File 2.** Table. Relative allocation of the dry mass of leaves, stolons and tillers to total shoots of 12 *B. humidicola* accessions (plus three checks: *B. brizantha*, *B. ruziziensis* and *B.* hybrid) grown under drained or waterlogged soil for 21 days. Data shown are means of six replicates ± SD. An asterisk represents significant differences between treatments for each accession (statistical significance at the *0.05, **0.01 and ***0.001 probability levels). NS: not significant. *P*_anova_ and LSD values exclude checks.

**File 3.** Table. Maximum rooting depth of 12 *B. humidicola* accessions (plus three checks: *B. brizantha*, *B. ruziziensis* and *B.* hybrid) before the beginning of the experiment. Data shown are means of six replicates ± SD. NS: not significant*. P*_anova_ and LSD values exclude checks.

**File 4.** Figure. Local inhibition of aerenchyma development by lateral roots. (A) Sequential cross-sections taken at ∼9.5–10.0 cm from the root tip of a nodal root of *B. humidicola* (CIAT 6707) grown under waterlogged conditions and (B) emergence of a lateral root from a nodal root (CIAT 16888) grown under waterlogged conditions. AE: air space; ST: stele; LR: lateral root. Scale bars = 250 µm.

Additional Information
