# Supplementary material for: Morpho-anatomical adaptations to waterlogging by germplasm accessions in a tropical forage grass
Source: AoB Plants. 2013 Nov 23;5:plt047. doi: 10.1093/aobpla/plt047 (PMC4455694; doi:10.1093/aobpla/plt047)
Supplement: Additional Information [file supp_5_plt047_index.html]

Morpho-anatomical adaptations to waterlogging by germplasm accessions in a tropical forage grass — Additional Information 

# Morpho-anatomical adaptations to waterlogging by germplasm accessions in a tropical forage grass

## Additional Information

Additional Information

**Files in this Data Supplement:**

- Additional Information Table 1 - doc file
- Additional Information Table 2 - doc file
- Additional Information Table 3 - doc file
- Additional Information Figure 1 - doc file
