# Supplementary material for: Morpho-anatomical adaptations to waterlogging by germplasm accessions in a tropical forage grass
Source: AoB Plants. 2013 Nov 23;5:plt047. doi: 10.1093/aobpla/plt047 (PMC4455694; doi:10.1093/aobpla/plt047)
Supplement: Additional Information [file supp_plt047_plt047supp_file1.doc]

**SUPPORTING INFORMATION**

**File 1. Table. Changes in leaf greenness (SPAD units) of 12 *B. humidicola* accessions (plus three checks: *B. brizantha*, *B. ruziziensis* and *B. hybrid*) grown under drained or waterlogged soil for 21 days.** Data shown are means of six replicates ± S.D. An asterisk (*) represents significant differences between treatments for each accession (statistical significance at the * 0.05, **0.01 and ***0.001 probability levels). All accessions showed a decrease of SPAD values under both treatments when compared to initial values (*P* < 0.05). *P* anova and LSD values exclude checks.

|  | Leaf greenness (SPAD units) | | | |
| --- | --- | --- | --- | --- |
|  | 0 days of treatment | | 21 days of treatment | |
| Accession | Drained | Waterlogged | Drained | Waterlogged |
| CIAT 26570 | 47.7 ± 4.1 | 44.4 ± 4.3 | 31.7 ± 3.0 | 28.9 ± 2.9 |
| CIAT 679 | 46.2 ± 2.2 | 45.6 ± 3.3 | 34.2 ± 6.1 | 30.5 ± 2.5 |
| CIAT 6133 | 43.0 ± 3.4 | 43.4 ± 4.7 | 36.7 ± 5.6 | 33.5 ± 4.5 |
| CIAT 16182 | 47.4 ± 4.0 | 44.5 ± 2.1 | 34.6 ± 3.7 | 29.5 ± 2.9 |
| CIAT 6707 | 39.2 ± 2.9 | 39.5 ± 5.0 | 29.3 ± 2.0 | 25.0 ± 2.8 |
| CIAT 16886 | 41.9 ± 3.8 | 42.3 ± 2.4 | 30.3 ± 3.5 | 29.8 ± 3.1 |
| CIAT 26152 | 49.9 ± 8.3 | 48.5 ± 5.7 | 34.1 ± 5.4 | 30.9 ± 4.8 |
| CIAT 6013 | 42.6 ± 3.3 | 45.0 ± 5.5 | 28.0 ± 4.1 | 26.9 ± 3.4 |
| CIAT 26416 | 41.8 ± 3.5 | 42.9 ± 2.1 | 29.2 ± 2.1 | 28.1 ± 4.6 |
| CIAT 26181 | 44.9 ± 4.9 | 45.5 ± 5.0 | 32.0 ± 1.1 | 31.7 ± 5.9 |
| CIAT 16866 | 44.5 ± 5.9 | 45.2 ± 3.4 | 33.1 ± 5.1 | 32.9 ± 5.1 |
| CIAT 16888 | 44.3 ± 5.5 | 41.2 ± 3.2 | 36.0 ± 3.1 | 31.7 ± 3.3 |
| *P* anova | 0.0081 | 0.0292 | 0.0037 | 0.0181 |
| LSD_0.05_ | 8.7 | 7.8 | 7.6 | 7.5 |
| Checks | | | | |
| *B. brizantha* | 40.6 ± 5.3 | 43.3 ± 6.0 | 33.4 ± 5.3 | 20.4 ± 2.7*** |
| *B. ruziziensis* | 49.7 ± 4.0 | 48.4 ± 4.2 | 30.8 ± 4.1 | 12.3 ± 3.2*** |
| *B*. hybrid | 43.0 ± 2.5 | 44.0 ± 3.9 | 32.3 ± 4.3 | 18.6 ± 3.1*** |
