# Supplementary material for: Morpho-anatomical adaptations to waterlogging by germplasm accessions in a tropical forage grass
Source: AoB Plants. 2013 Nov 23;5:plt047. doi: 10.1093/aobpla/plt047 (PMC4455694; doi:10.1093/aobpla/plt047)
Supplement: Additional Information [file supp_plt047_plt047supp_file2.doc]

**SUPPORTING INFORMATION**

**File 2. Table**. **Relative allocation of dry mass of leaves, stolons and tillers to total shoot of 12 *B. humidicola* accessions (plus three checks: *B. brizantha*, *B. ruziziensis* and *B.* hybrid) grown under drained or waterlogged soil for 21 days.** Data shown are means of six replicates ± S.D. An asterisk (*) represents significant differences between treatments for each accession (statistical significance at the * 0.05, ** 0.01 and *** 0.001 probability levels). N.S.: Not significant. *P* anova and LSD values exclude checks.

|  | Ratio to total shoot | | | | | |
| --- | --- | --- | --- | --- | --- | --- |
|  | Leaves | | Stolons | | Tillers | |
| Accession | Drained | Waterlogged | Drained | Waterlogged | Drained | Waterlogged |
| CIAT 26570 | 26.9 ± 4.8 | 23.4 ± 3.1 | 48.5 ± 24.6 | 60.1 ± 16.5 | 24.6 ± 2.0 | 16.5 ± 0.8*** |
| CIAT 679 | 19.0 ± 2.0 | 16.4 ± 1.3** | 52.6 ± 28.4 | 65.2 ± 18.4 | 28.4 ± 2.7 | 18.4 ± 1.1*** |
| CIAT 6133 | 23.9 ± 2.4 | 23.1 ± 3.3 | 50.6 ± 25.5 | 59.9 ± 17.0 | 25.5 ± 1.7 | 17.0 ± 1.4*** |
| CIAT 16182 | 22.5 ± 2.5 | 19.6 ± 6.2 | 50.5 ± 27.1 | 61.7 ± 18.7 | 27.1 ± 3.4 | 18.7 ± 3.1*** |
| CIAT 6707 | 27.6 ± 5.0 | 19.2 ± 2.9** | 46.9 ± 25.5 | 63.0 ± 17.0 | 25.5 ± 2.4 | 17.8 ± 1.2*** |
| CIAT 16886 | 39.5 ± 3.3 | 26.2 ± 4.8*** | 38.6 ± 21.9 | 55.0 ± 18.8 | 21.9 ±2.9 | 18.8 ± 2.7 |
| CIAT 26152 | 27.0 ± 3.1 | 17.4 ± 3.2*** | 48.4 ± 24.6 | 63.4 ± 19.1 | 24.6 ± 1.7 | 19.1 ± 2.5*** |
| CIAT 6013 | 32.0 ± 11.4 | 18.3 ± 2.8** | 41.8 ± 26.2 | 62.3 ± 19.4 | 26.2 ± 7.4 | 19.4 ± 3.6 |
| CIAT 26416 | 25.1 ± 6.7 | 19.1 ± 19.0 | 50.5 ± 24.4 | 63.3 ± 17.6 | 24.4 ± 1.8 | 17.6 ± 1.3*** |
| CIAT 26181 | 26.9 ± 6.8 | 19.5 ± 1.9* | 46.5 ± 26.6 | 61.0 ± 19.5 | 26.6 ± 4.8 | 19.5 ± 1.4** |
| CIAT 16866 | 33.4 ± 5.3 | 31.3 ± 1.5 | 45.0 ± 21.6 | 48.4 ± 20.3 | 21.6 ± 1.2 | 20.3 ± 4.4 |
| CIAT 16888 | 40.3 ± 5.9 | 38.2 ± 6.1 | 39.5 ± 20.2 | 42.0 ± 19.8 | 20.2 ± 2.3 | 19.8 ± 2.8 |
| *P* anova | 0.0000 | 0.0000 | 0.9971 | 0.5961 | 0.0020 | 0.1206 |
| LSD_0.05_ | 10.5 | 7.1 | N.S. | N.S. | 6.2 | N.S. |
| Checks | | | | | | |
| *B. brizantha* | 64.5 ± 3.5 | 57.2 ± 11.7 | - | - | 35.5 ± 3.5 | 42.8 ± 11.7 |
| *B. ruziziensis* | 41.4 ± 3.4 | 30.2 ± 3.8*** | - | - | 58.6 ± 3.4 | 69.8 ± 3.8 |
| *B*. hybrid | 56.9 ± 7.5 | 55.2 ± 9.5 | - | - | 43.1 ± 7.5 | 44.8 ± 9.5 |
