# Supplementary material for: Morpho-anatomical adaptations to waterlogging by germplasm accessions in a tropical forage grass
Source: AoB Plants. 2013 Nov 23;5:plt047. doi: 10.1093/aobpla/plt047 (PMC4455694; doi:10.1093/aobpla/plt047)
Supplement: Additional Information [file supp_plt047_plt047supp_file3.doc]

**SUPPORTING INFORMATION**

**File 3. Table. Maximum rooting depth of 12 *B. humidicola* accessions (plus three checks: *B. brizantha*, *B. ruziziensis* and *B. hybrid*) before the beginning of the experiment.** Data shown are means of six replicates ± S.D. N.S.: Not significant*. P* anova and LSD values exclude checks.

|  | Maximum rooting depth (cm plant^-1^) | |
| --- | --- | --- |
|  | 0 days of treatment | |
| Accession | Drained | Waterlogged |
| CIAT 26570 | 27.0 ± 9.8 | 24.3 ± 7.1 |
| CIAT 679 | 24.0 ± 7.8 | 25.7 ± 13.2 |
| CIAT 6133 | 25.0 ± 12.8 | 23.2 ± 9.3 |
| CIAT 16182 | 32.2 ± 15.0 | 30.2 ± 15.2 |
| CIAT 6707 | 29.3 ± 6.8 | 25.3 ± 12.7 |
| CIAT 16886 | 23.5 ± 8.7 | 27.2 ± 9.9 |
| CIAT 26152 | 33.2 ± 7.9 | 29.3 ± 8.5 |
| CIAT 6013 | 33.0 ± 13.2 | 37.5 ± 9.1 |
| CIAT 26416 | 23.3 ± 12.5 | 23.0 ± 8.2 |
| CIAT 26181 | 26.5 ± 10.0 | 25.2 ± 12.3 |
| CIAT 16866 | 25.0 ± 9.9 | 26.0 ± 8.6 |
| CIAT 16888 | 20.0 ± 6.7 | 25.8 ± 9.3 |
| *P* anova | 0.4594 | 0.5911 |
| LSD_0.05_ | N.S. | N.S. |
| Checks | | |
| *B. brizantha* | 51.2 ± 9.4 | 42.2 ± 23.1 |
| *B. ruziziensis* | 30.5 ± 7.1 | 36.8 ± 11.7 |
| *B*. hybrid | 40.3 ± 12.0 | 32.5 ± 16.5 |
