# Supplementary material for: Morpho-anatomical adaptations to waterlogging by germplasm accessions in a tropical forage grass
Source: AoB Plants. 2013 Nov 23;5:plt047. doi: 10.1093/aobpla/plt047 (PMC4455694; doi:10.1093/aobpla/plt047)

**SUPPORTING INFORMATION**

**File 4.** Figure. Local inhibition of aerenchyma development by lateral root. (A) Sequential cross sections taken at ~ 9.5-10.0 cm from the root tip of a nodal root of *B. humidicola* (CIAT 6707) grown under waterlogged conditions and (B) emergence of a lateral root from a nodal root (CIAT 16888) grown under waterlogged conditions. AE: air space; ST: stele; LR: lateral root. Scale bars equal to 250 µm.


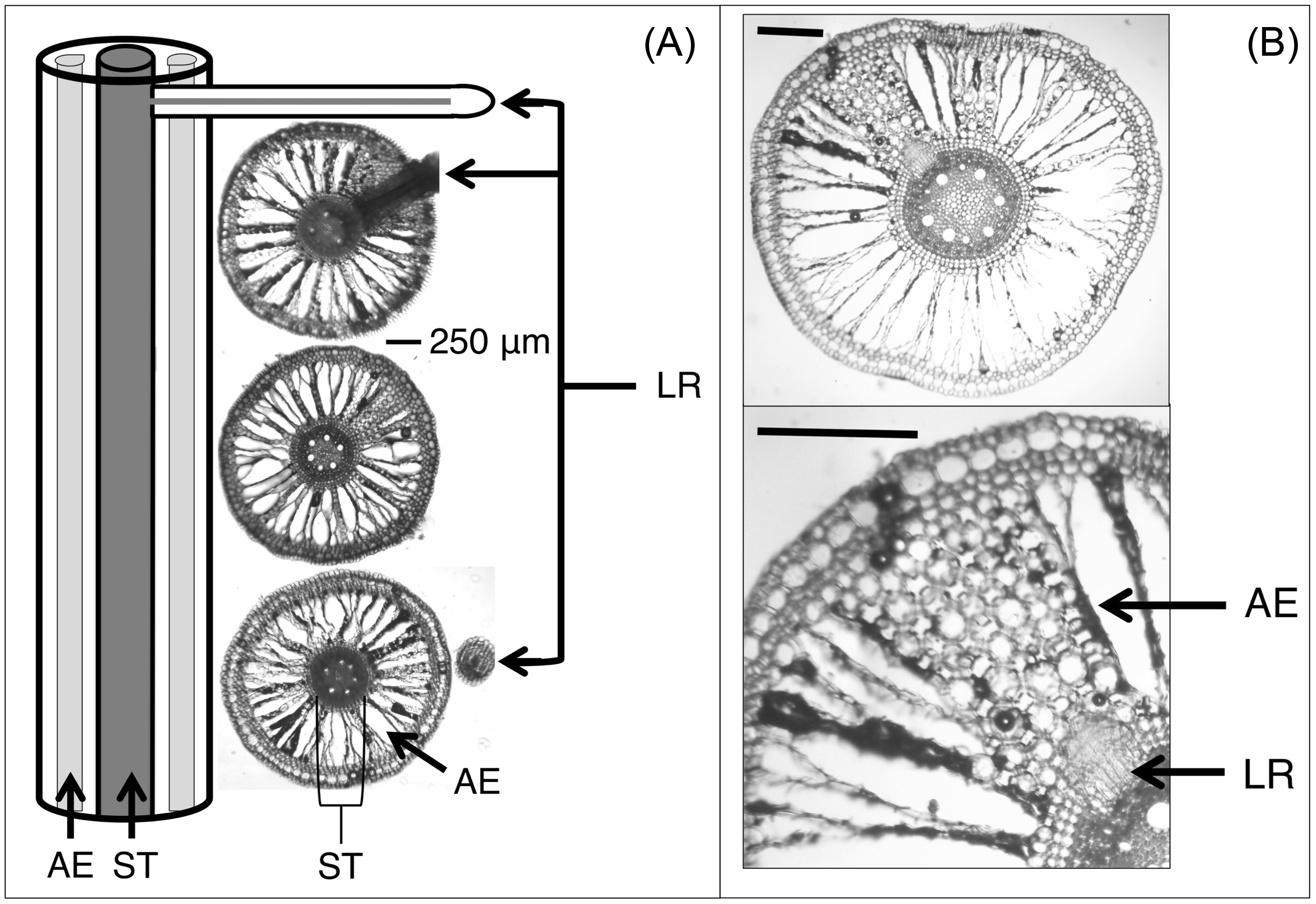

Supplement: Additional Information [file supp_plt047_plt047supp_file4.doc]
